# Clinically determined type of ^18^F-fluoro-2-deoxyglucose uptake as an alternative prognostic marker in resectable pancreatic cancer

**DOI:** 10.1371/journal.pone.0172606

**Published:** 2017-02-24

**Authors:** Jae Uk Chong, Ho Kyoung Hwang, Jin Ho Lee, Mijin Yun, Chang Moo Kang, Woo Jung Lee

**Affiliations:** 1 Division of Hepatobiliary and Pancreatic Surgery, Department of Surgery, Yonsei University College of Medicine, Seoul, Korea; 2 Pancreaticobiliary Cancer Clinic, Yonsei Cancer Center, Severance Hospital, Seoul, Korea; 3 Department of Surgery, National Health Insurance Corporation Ilsan Hospital, Goyang, Korea; 4 Department of Nuclear Medicine, Yonsei University College of Medicine, Seoul, Korea; University of Chicago, UNITED STATES

## Abstract

**Purpose:**

To investigate the association between clinical PET (positron emission tomography) type and oncologic outcome in resectable pancreatic cancer.

**Methods:**

Between January 2008 and October 2012, patients who underwent potentially curative resection for resectable pancreatic ductal adenocarcinoma without neoadjuvant treatment were retrospectively investigated. Clinical PET type was defined as follows: pancreatic cancer with similar ^18^FDG uptake to renal calyx was determined as kidney-type (K-type), and relatively lower ^18^FDG uptake than that of renal calyx was regarded as Non-K type.

**Results:**

A total of 53 patients were enrolled. After agreement-based reclassification, agreement based K-type (aK-type) was noted in 34 patients (64.2%), and agreement based Non-K type (aNon K-type) was found in 19 patients (35.8%). There was a significant difference between aK-type and aNon K-type pancreatic cancer (tumor size (*P* = 0.030), adjusted CA 19–9 (*P* = 0.007), maximum standard uptake value (SUV_max_,*P*<0.001), metabolic tumor volume (MTV_2.5_, *P*<0.001), total lesion glycolysis (TLG, *P*<0.001)). K-type pancreatic cancer (n = 31) showed a significantly shorter disease-free time compared with Non-K type (n = 16) (10.8 vs. 24.1 months, *P* = 0.013). It was also noted that aK-type showed inferior disease-free survival to that of aNon-K type pancreatic cancer (11.9 vs. 28.6 months, *P* = 0.012).

**Conclusions:**

Clinical PET type is a reliable clinical marker to estimate aggressive tumor biology and can be utilized in predicting tumor recurrence and necessity for postoperative chemotherapy.

## Introduction

^18^F-fluoro-2-deoxyglucose (^18^F-FDG) positron emission tomography/computed tomography (PET/CT) scan is the functional imaging technology used to detect tumors with a high metabolic rate. It can also provide quantification of metabolic activity such as maximum standard uptake value (SUV_max_), metabolic tumor volume (MTV), and total lesion glycolysis (TLG) for estimating tumor biology and clinical translation [1,2].

In general, overexpression of glucose transporters and hexokinases has been reported in many cancer cells[3]. ^18^F-FDG is taken up by up-regulated surface glucose transporters and is phosphorylated by hexokinases. Glucose-6-phosphatase dephosphorylates glucose (FDG) to participate in the normal metabolic process. However, cancer cells have low expression of glucose-6-phosphatase compared to many normal tissues, and this can lead to an accumulation of ^18^F-FDG-P in tumor cells[4–6]. The ^18^F-FDG-PET scan is currently being used for cancer diagnosis[7], staging[8], identifying hidden metastasis, and assessment of treatment responses[9] in clinical oncology.

There have been several studies showing the oncologic significance of ^18^F-FDG-PET scans in predicting prognosis in pancreatic cancer [10–12]. Specifically, Dholakia et al[13] recently reported that MTV and TLG are significant prognostic factors of overall survival in patients with locally advanced pancreatic cancer. Epelbaum et al. [14] assessed the role of a quantitative dynamic PET model in pancreatic cancer and concluded that global ^18^F-FDG influx was the most important parameter to predict overall survival. Yamamoto et al. [15] evaluated the clinical usefulness of ^18^F-FDG-PET scans as a prognostic marker in resected pancreatic cancer and found that an SUV_max_ greater than 6.0 was a significant predictor of early postoperative recurrence and poor survival in resected pancreatic cancer.

Although there are important studies suggesting potential associations between PET-based parameters and oncologic outcomes, the calculation and official documentation of individual PET-based parameters might not be routine in clinical practice because these processes usually require time- and labor-consuming processes for the radiologists. In addition, these parameters are somewhat subjective and prone to observer variability [16].

Interestingly, some studies have shown a potential relationship between image-based interpretation of tumors and oncologic outcome in treating malignant disease [17–21]. In this study, we analyzed the clinical feasibility of quick “qualitative” assessment of FDG-uptake in resectable pancreatic cancer by surgeons. We tried to correlate this clinical PET type with clinicopathological characteristic and oncologic outcome in resected pancreatic cancer. The goal of this study was to propose a qualitatively assessed clinical PET-type method that can be an alternative prognostic marker in resectable pancreatic cancer.

## Materials and methods

### Patient selection and clinicopathologic characteristics

We retrospectively reviewed medical records of patients who underwent potentially curative resection for resectable pancreatic ductal adenocarcinoma. Only patients who underwent surgical resection with preoperative ^18^F-FDG PET/CT as part of a staging work-up between January 2008 and October 2012 were included. Unresectable locally advanced pancreatic cancer and metastatic pancreatic cancer were excluded. In addition, those who received preoperative neoadjuvant treatment for borderline or locally advanced pancreatic cancer on preoperative imaging modalities were excluded due to potential impact of neoadjuvant treatment [22]. The study was reviewed and approved by the Institutional Review Board of Yonsei University College of Medicine.

The variables of gender, age, tumor location, operation type, tumor size, preoperative serum CA 19–9 (actual CA 19–9), adjusted CA 19–9 (calculated as actual CA 19–9 divided by initial serum bilirubin), grade (differentiation), pathologic tumor (pT) stage, presence of lymph node metastasis (pN), lymph node ratio (total number of metastatic lymph nodes divided by total number of retrieved lymph nodes), retrieved number of LNs, number of metastastic LNs, microscopic perineural invasion, lymphovascular invasion, recurrence, and time to recurrence were retrospectively reviewed. Maximum standard uptake value (SUV_max_), metabolic tumor volume (MTV_2.5_), and total lesion glycolysis (TLG) were measured by two nuclear medicine physicians as described previously [10,23]. Each tumor was examined with a spherical-shaped volume of interest (VOI). SUV_max_ of the VOI was calculated as (decay-corrected activity/tissue volume)/(injected dose/body weight). MTV_2.5_ was defined as total tumor volume with an SUV of 2.5 or greater. TLG was calculated as (mean SUV) x (MTV_2.5_). In order to assess the possible influence of renal function on FDG uptake, estimated glomerular filtration rate (eGFR) and serum creatinine (Cr) levels were also reviewed.

### Determining clinical PET type

**Fig 1:**
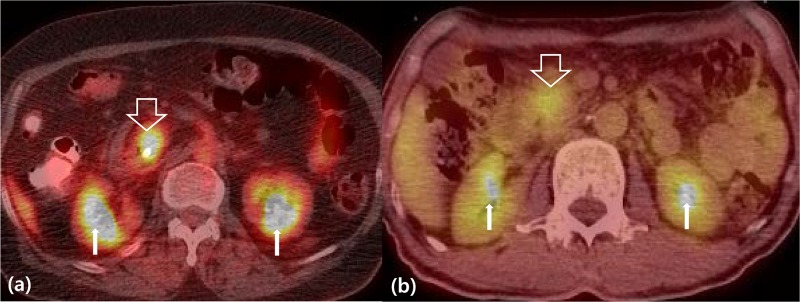
Determining clinical PET type based on perceived FDG-uptake intensity in the renal calyx. (a) K-type, the perceived signal intensity of FDG-uptake in pancreatic head cancer (thick empty white arrow) is similar to that of the renal calyx(thin white arrow) (b) Non-K-type, the perceived signal intensity of FDG-uptake in pancreatic head cancer (thick empty white arrow) is lower than that of the renal calyx(thin white arrow).

Perceived signal intensity of ^18^FDG in the renal calyceal system was used as a reference to categorize clinical PET type. Pancreatic cancer with similar ^18^FDG uptake to that of the renal calyx was determined as K-type (Fig 1a), and pancreatic cancer with relatively lower ^18^FDG uptake than that of renal calyx was regarded as Non-K-type (Fig 1b). Three surgeons (Kang CM, Hwang HK, Lee JH) were asked to categorize the patients according to this defining system for clinical PET type. During the process of individual classification, the surgeons were not allowed to communicate regarding their interim results. However, the respectively determined clinical PET types were re-categorized as aK-type and aNon-K-type based on surgeon agreement. Agreement-based reclassification of clinical PET type follows the agreed upon classification of two surgeons. For example, if two surgeons determined a sample to be K-type and one surgeon concluded Non-K-type, the agreement-based reclassification of clinical PET type would be aK-type.

### Statistical analysis

Continuous variables were described as mean ± standard deviation, and categorical variables were described as frequency (%). Student’s t-test was used for these determinations. To estimate inter-observer discrepancy, average agreement and Cohen’s Kappa values were analyzed, and results were defined as follows: poor < 0.2, fair 0.21–0.4, moderate 0.41–0.6, substantial 0.61–0.8, and excellent 0.81–1[24]. Survival curves were estimated using the Kaplan-Meier method to calculate cumulative disease-free survival. Statistical analyses were performed using SPSS 20.0 for Windows (SPSS Inc., Chicago, IL, USA). *P*-values<0.05 were considered statistically significant.

## Results

### Patient demographics and defining clinical PET type in resectable pancreatic cancer

**Fig 2:**
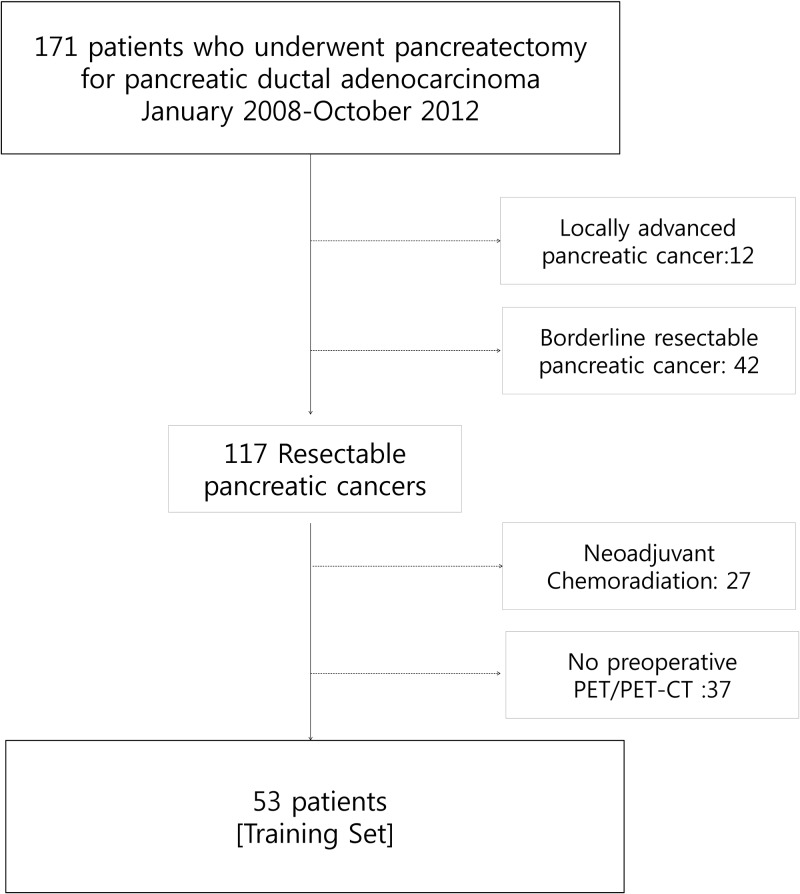
Patient eligibility.

A total of 53 patients were enrolled for this study (Fig 2). The clinicopathological characteristics are summarized in Table 1. The mean disease-free survival was 20.8 months [95% CI: 15.6–26.1], and mean disease-specific survival was 30.5 months [95% CI: 24.3–36.7]. With regard to clinical PET type, 31 patients (58.5%) were found to have K-type, and 16 patients (31.2%) were Non-K-type. The other six patients (11.3%) had cancer whose type was unable to be agreed upon by all surgeons. In terms of renal function, all patients had normal serum Cr levels. However, 26 patients (49%) had eGFR below 90 mL/min/1.73m^2^ with mild to moderate decrease [25]. There was no significant correlation between eGFR and SUV_max_ in patient with normal serum creatinine levels (r = -0.115, *P* = 0.441).

**Table 1:** Clinicopathological characteristics of the patients.

Variables	Frequency, Mean ± SD
Age (years)	63.1 ± 9.2
Gender (Female/Male)	24/29
Tumor Size, cm	2.3 ± 0.7
Location (Head/Body/Tail)	38/12/3
CA 19–9, U/mL	509.6 ± 1675.9
PD(PPPD)/DPS/TP	6(32)/12/3
Grade (W/M/P/U)	8/39/6
T stage (T1/T2/T3)	2/2/49
N stage (N0/N1)	24/29
Retrieved LNs	18.3 ± 7.7
Metastatic LNs	1.3 ± 2.2
LNR	0.08 ± 0.11
PNI (No/Yes)	14/39
LVI (No/Yes)	34/19
R0/R1/R2	53/0/0
SUV_max_	5.3 ± 2.8
MTV_2.5_	3.9 ± 3.8
TLG	16.9 ± 20.9
eGFR	91.9 ± 18.9
Serum Cr	0.80 ± 0.20

PD, pancreaticoduodenectomy; PPPD, pylorus preserving pancreaticoduodenectomy; Grade (W/M/P/U), well-, moderate-, poor-, un-differentiated; LNs, lymph nodes; LNR, lymph node ratio; PNI, perineural invasion; LVI, lymphovascular invasion; SUV_max_, maximum standard uptake value; MTV_2.5,_ metabolic tumor volume; TLG, total lesion glycolysis; eGFR, estimated glomerular filtration rate; Cr, creatinine

### Correlations between clinicopathological characteristics and clinical PET type in resectable pancreatic cancer

There were no significant differences between K-type and Non-K-type pancreatic cancer in terms of clinicopathological characteristics such as tumor size, pT-stage, pN-stage, lymphovascular invasion, perineural invasion, and tumor differentiation (*P*>0.05). However, SUV_max_ (*P*<0.001), MTV_2.5_ (*P*<0.001) and TLG (*P*<0.001) were found to be statistically different between K-type and Non-K-type pancreatic cancer as determined by individual surgeons. Specifically, the actual CA 19–9 level appeared to be higher in K-type pancreatic cancer, but the difference was not significant (*P*>0.05). However, the adjusted CA 19–9 was significantly different between two surgeons, S2 (448.6 ± 62.2 vs. 98.6 ± 153.9, *P* = 0.006) and S3 (434.8 ± 656.9 vs. 104.9 ± 156.3, *P* = 0.008, Table 2).

**Table 2:** Clinicopathological differences according to individual surgeons’ clinical type of FDG-uptake.

	S1		S2		S3	
	K	Non-K	K	Non-K	K	Non-K
	(N = 34)	(N = 19)	(N = 33)	(N = 20)	(N = 34)	(N = 19)
**Age, years**	62.4 ± 9.9	64.4 ± 7.6	62.5 ± 9.9	64.1 ± 7.9	62.4 ± 9.9	64.7 ± 7.8
**Gender (Female/Male)**	15/19	09/10	14/19	10/10	14/20	10/9
**Tumor Size, cm**	2.5 ± 0.7	2.3 ± 0.8	2.5 ± 0.7	2.2 ± 0.8	2.5 ± 0.7	2.2 ± 0.8
**Location (Head/Body/Tail)**	22/9/3	16/3/0	23/8/2	15/4/1	23/8/3	15/4/0
**Actual CA 19–9, U/mL**	670.8 ± 2077.9	221.1 ± 245.6	703.7 ± 2104.2	189.2 ± 237.2	677.1 ± 2075.6	209.9 ± 260.8
**Adjusted CA 19–9, U/mL**	390.8 ± 625.1	183.5 ± 379.4	448.6 ± 62.2	98.6 ± 153.9^a^	434.8 ± 656.9	104.9 ± 156.3^b^
**Grade (W/M/P/U)**	3/27/4	5/12/2	4/25/4	4/14/2	4/26/4	4/13/2
**T stage (T1/T2/T3)**	3/2/29	2/2/15	1/2/30	1/0/19	1/2/31	1/0/18
**N stage (N0/N1)**	15/19	9/10	14/19	10/10	14/10	10/9
**Retrieved LNs**	17.2 ± 6.9	20.3 ± 8.6	17.7 ± 7.9	19.4 ± 8.6	17.4 ± 7.1	20.0 ± 8.6
**Metastatic LNs**	1.3 ± 2.5	1.4 ± 1.6	1.3 ± 2.5	1.4 ± 1.6	1.3 ± 2.5	1.4 ± 1.6
**LNR**	0.08 ± 0.11	0.09 ± 0.11	0.07 ± 0.11	0.08 ± 0.11	0.07 ± 0.11	0.08 ± 0.11
**PNI (No/Yes)**	10/24	4/15	7/26	7/13	8/26	6/13
**LVI (No/Yes)**	22/12	12/7	22/11	12/8	22/12	12/7
**R0/R1/R2**	34/0/0	19/0/0	33/0/0	20/0/0	34/0/0	19/0/0
**SUV**_**max**_	6.3 ± 2.9	3.2 ± 0.6^c^	6.3 ± 2.9	3.2 ± 0.6^d^	6.2 ± 2.9	3.1 ± 0.6^e^
**MTV**_**2.5**_	5.5 ± 3.7	0.8 ± 0.9^c^	5.3 ± 3.8	1.1 ± 1.6^d^	5.4 ± 3.7	0.7 ± 0.9^e^
**TLG**	23.9 ± 22.5	3.0 ± 3.7^c^	23.7 ± 22.9	4.4 ± 6.7^d^	23.8 ± 22.5	3.0 ± 4.1^e^
**Serum Cr**	0.79 ± 0.19	0.81 ± 0.22	0.79 ± 0.19	0.81 ± 0.21	0.80 ± 0.19	0.80 ± 0.21
**eGFR**	91.4 ± 17.8	92.7 ± 21.2	91.5 ± 17.9	92.5 ± 20.9	92.2 ± 18.3	91.2 ± 20.3

S1, surgeon1; S2, surgeon2; S3, surgeon3; Grade (W/M/P/U), well-, moderate-, poor-, un-differentiated; LNs, lymph nodes; LNR, lymph node ratio; PNI, perineural invasion; LVI, lymphovascular invasion; SUV_max_, maximum standard uptake value; MTV_2.5,_ metabolic tumor volume; TLG, total lesion glycolysis; eGFR, estimated glomerular filtration rate; Cr, creatinine.

^a^ *P* = 0.006,

^b^ *P* = 0.008,

^c,d,e^ *P*<0.001

### Analysis of inter-surgeon agreement

Overall, the inter-surgeon agreement was greater than 91% with a pairwise Cohen’s kappa of 0.81 (Table 3). After agreement-based reclassification, aK-type was noted in 34 patients (64.2%), and aNon-K-type pancreatic cancer was found in 19 patients (35.8%, Table 4). There were significant statistical differences between agreement-based aNon-K-type and aK-type in adjusted CA 19–9 (102.8 ± 156.9 vs. 435.9 ± 656.3, *P* = 0.007), tumor size (2.0 ± 0.4 vs. 2.5 ± 0.7, *P* = 0.030), SUV_max_ (3.1 ± 0.7 vs. 6.2 ± 2.8, *P*<0.001), MTV_2.5_ (1.0 ± 1.4 vs. 5.9 ± 4.6, *P*<0.001), and TLG (3.2 ± 4.1 vs. 23.8 ± 22.6, *P*<0.001).

**Table 3:** Inter-surgeon discrepancy.

	S1 and S2	S2 and S3	S3 and S1	Average value
Pairwise percent agreement (%)	88.679	90.566	94.34	91.195
Pairwise Cohen’s Kappa	0.752	0.797	0.878	0.81

S1, surgeon1; S2, surgeon2; S3, surgeon3

**Table 4:** Agreement-based clinical PET type in six patients in whom not all three surgeons agreed on PET type.

Patient Number	S1	S2	S3	Agreement-based decision^1^
1	K-type	Non K-type	Non K-type	aNon-K-type
2	Non K-type	Non K-type	K-type	aNon-K-type
3	Non K-type	K-type	K-type	aK-type
4	Non K-type	K-type	K-type	aK-type
5	K-type	Non K-type	Non K-type	aNon-K-type
6	K-type	K-type	Non K-type	aK-type

S1, surgeon1; S2, surgeon2; S3, surgeon3

^1^Agreement-based decision of clinical PET type follows the agreed upon classification of at least two surgeons

### Oncologic outcome according to clinical PET type in resectable pancreatic cancer

It was found that preoperatively determined clinical PET type could predict tumor recurrence after radical pancreatectomy. There was a significant difference in disease-free survival between individually determined K-type and Non-K-type resected pancreatic cancer (*P*<0.05, Table 5).

**Table 5:** Disease-free survival according to clinical PET type determined by individual surgeons.

	K-type	Non-K-type	P-value
S1	17.6 months [95% CI:11.2–23.9]	24.5 months [95% CI:17.7–31.4]	0.035
S2	11.4 months [95% CI: 8.6–14.3]	29.3 months [95% CI: 21.1–37.5]	0.003
S3	11.9 months [95% CI: 9.1–14.7]	29.6 months [95% CI: 20.9–38.2]	0.007

S1, surgeon1; S2, surgeon2; S3, surgeon3; CI, confidence interval

When analyzing oncologic outcomes according to agreement of all three surgeons, K-type (N = 31, mean disease-free survival, 10.8 months [95% CI: 8.3–13.3]) showed significant early recurrence compared with Non-K-type pancreatic cancer (N = 16, mean disease-free survival, 24.1 months [95% CI: 24.4–54.8], *P* = 0.013). Disease-free survival of six patients with disagreed clinical type of ^18^FDG uptake among the surgeons showed similar oncologic outcomes to Non-K-type patients (*P* = 0.237).

**Fig 3:**
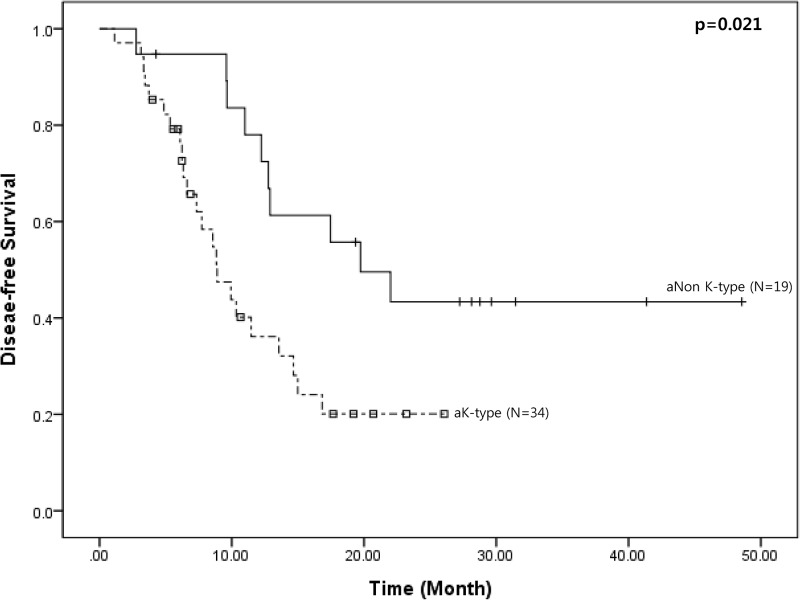
Oncologic outcomes according to agreement-based reclassification of clinical PET type. aK-type, agreement-based K-type; aNon K-type, agreement-based Non K-type.

Finally, according to agreement-based reclassification, aK-type showed inferior mean disease-free survival compared to aNon-K-type pancreatic cancer (11.9 months [95% CI: 9.0–14.9] vs. 28.6 months [95% CI: 20.2–36.9], *P* = 0.012, Fig 3).

### Oncologic impact of postoperative chemotherapy according to clinical PET type in resectable pancreatic cancer

**Fig 4:**
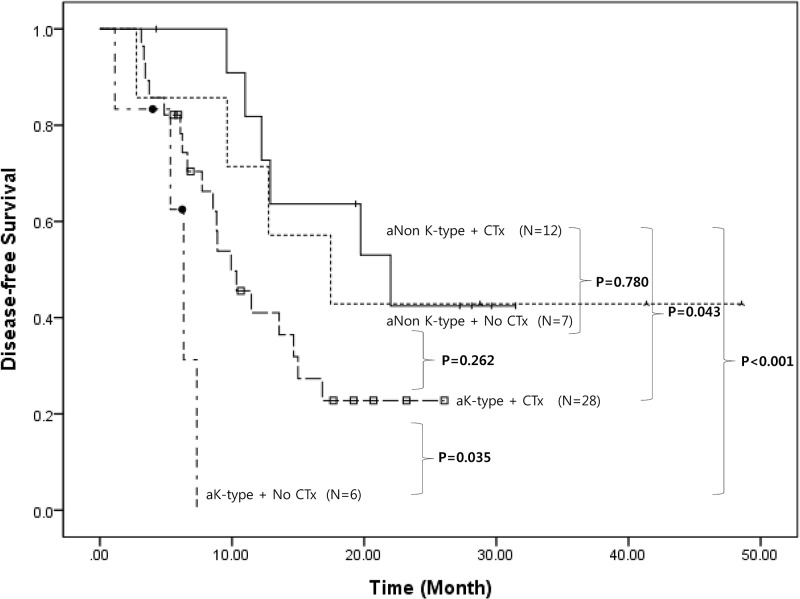
Oncologic role of postoperative chemotherapy according to clinical PET type in resectable pancreatic cancer. aK-type, agreement-based K-type; aNon K-type, agreement-based Non K-type; CTx, postoperative chemotherapy.

Postoperative chemotherapy was offered to all patients after curative resection. However, 13 patients (25%) were not able to receive postoperative chemotherapy. Among these patients, 5 patients (38%) refused further treatment, 3 patients (24%) developed postoperative complications preventing postoperative chemotherapy in a timely manner, and remaining 5 patients (38%) did not recover well-enough for further treatment. There was no significant oncologic impact of postoperative chemotherapy on patients with aNon-K-type pancreatic cancer (mean 26.9 months [95% CI: 12.7–41.1] vs. 21.9 months [16.6–27.3], *P* = 0.780). However, postoperative chemotherapy played a very important role in patients with aK-type pancreatic cancer. In patients with aK-type pancreatic cancer, disease-free survival improved with postoperative chemotherapy (mean 5.6 months [95% CI: 3.6–7.6] vs. mean 12.8 months [95% CI: 9.7–16.0], *P* = 0.035), leading to comparable oncologic outcomes with aNon-K-type without postoperative chemotherapy (*P* = 0.262). Results of univariate and multivariate analysis of disease-free survival for aK-type have also revealed that postoperative chemotherapy is an independent prognostic factor in recurrence (HR 0.290, 95% CI: 0.086–0984, *P* = 0.047, Table 6). However, postoperative chemotherapy still could not improve disease-free survival to the extent of aNon-K-type pancreatic cancer with postoperative chemotherapy (*P* = 0.043, Fig 4).

**Table 6:** Univariate and multivariate analysis of disease-free survival for aK-type.

Variables	N = 34 (%)	Univariate analysis	Multivariate analysis	
		p-value	p-value	HR (95%CI)
Age > 65 years	15 (44)	0.309		
Male gender	20 (59)	0.058		
ASA score		0.591		
1	11 (32)			
2	20 (59)			
3	3 (9)			
Tumor size ≥ 2.5cm	21 (62)	0.802		
AJCC 7^th^ stage		0.960		
I/IIA	14 (41)			
IIB	20 (59)			
Postoperative chemotherapy	28 (82)	0.035	0.047	0.290 (0.086-0.984)

## Discussion

^18^F-fluoro-2-deoxyglucose (^18^F-FDG) positron emission tomography/computed tomography (PET/CT) is an emerging radiologic technique to detect functional (metabolic and biologic) properties of cancer [26,27]. ^18^F-FDG-PET/CT is a potential preoperative image modality because tumor biology can be estimated using PET-based parameters even in preoperative staging situations.

In this study, we defined a qualitatively assessed clinical PET type and correlated it with oncologic outcome of resected pancreatic cancer. Pancreatic cancers with an available preoperative PET-scan could be divided into K-type and Non-K-type according to perceived signal intensity of ^18^F-FDG uptake compared to that shown in the renal calyx. There are other potential candidates for a reference organ for determining the signal of ^18^FDG uptake such as brain and myocardium. However, to compare the signal intensity of FDG uptake in a pancreatic tumor, one would need to frequently move the axial section-field to the chest level or even the brain area, which would be inconvenient during clinical assessment of metabolic properties in pancreatic cancer. On the contrary, the renal calyx can be easily visualized due to its proximity to the pancreas, allowing it to be easily used as a reference organ in determining clinical PET type in resected pancreatic cancer (Fig 1). Perceived intensity of FDG uptake in renal calyx may be different among patients. However, clinical PET typing is derived from comparison of perceived intensity of renal calyx and pancreatic cancer within the patient. This can be achieved easily in a single image with use of renal calyx as reference, which is in proximity to pancreas.

According to our results, the clinical PET type (K-type and Non-K-type) was found to successfully discriminate disease-free survival in resected pancreatic cancer (*P*<0.05, Fig 3 and Table 5). Results of our finding concur with previously reported association between SUV_max_ greater than 6.0 with early postoperative recurrence following resection of pancreatic cancer [15]. The present clinical PET type is based on surgeons’ perceptions of ^18^FDG uptake in the tumor, with the renal calyx as the reference signal. Therefore, it is thought that clinical PET type can be very subjective and so might not be reliable. However, unlike our expectations, the agreement rate among three individual surgeons was estimated to be higher than 91.195% with a pairwise Cohen’s Kappa value of 0.81, suggesting excellent inter-observer variability. In previous reports, interobserver variation for SUV_mean_ measurement has been noted up to 17% [28] and interobserver agreement for SUV_max_ has been reported to be 91–93% [29]. This means that application of clinical PET type can be reliably used as an alternative detectable parameter to estimate tumor glucose metabolism and tumor biology in clinical setting.

Our data also showed a predictive value for tumor recurrence of clinical PET type, suggesting it as a potential clinical biomarker to predict recurrence before surgical intervention, especially when PET-based parameters are not documented in clinical practice. Biological mechanism behind our results may be explained by the role of SUV_max_ in PET imaging. Kang et al. [30] reported that loss of SMAD4 is associated with poor oncologic outcome and this was correlated with SUV_max_ to conclude that higher SUV_max_ was associated with loss of SMAD4. Our results have shown that K-types have higher SUV_max_ (Table 2). This may explain poor disease-free survival of aK-type patients. Further research on role of PET imaging in prognosis of pancreatic cancer is needed.

There are several other advantages of the current qualitative method of determination of clinical PET type. First, the current system for determining clinical PET type is simple, easy, reproducible, and practical. Without specialized effort and equipment to measure PET-based parameters, surgeons or clinicians can estimate oncologic outcome during consultations with patients who have had a preoperative PET scan on spot.

Second, in spite of surgeons’ subjective determination, PET-based parameters of SUV_max_, MTV, and TLG were significantly different between K-type and Non-K-type pancreatic cancer (Table 4). In addition, when correlating clinical PET type and preoperative serum CA 19–9, our data showed a higher level of preoperative actual CA 19–9, but the difference was not statistically significant. However, the adjusted CA 19–9 was found to be correlated with clinical PET type for surgeons S2 and S3, suggesting that clinical PET type can be a useful preoperative prognostic marker in resectable pancreatic cancer (Table 2). These observations should be confirmed when analyzing data according to agreement-based clinical PET type.

Some studies have also suggested potential association between CA 19–9 and PET-based parameters. Shi et al[31] showed that MTV and TLG were most strongly correlated with serum CA 19–9 in patients with resected pancreatic cancer. Xu et al[12] also observed that MTV and TLG were significantly associated with baseline serum CA 19–9, and MTV and TLG showed strong consistency with baseline serum CA 19–9, leading to improved predictions of oncologic outcome in resectable pancreatic cancer. In fact, we have already studied the oncologic significance of adjusted CA 19–9 in predicting tumor recurrence in resected pancreatic cancer [32]. In the current data set, when setting the cut-off value of adjusted CA 19–9 to 80, we were able to predict disease-free survival in resected pancreatic cancer (*P* = 0.044, data not shown). Using a larger study volume, it will be necessary to validate this potential relationship between clinical PET type and serum CA 19–9 in the near future.

Finally, it was shown that preoperatively determined clinical PET type, especially, K-type pancreatic cancer, requires postoperative chemotherapy after radical pancreatectomy. According to our data, disease-free survival of aK-type pancreatic cancer is influenced by postoperative adjuvant chemotherapy (Fig 3), suggesting that aK-type resectable pancreatic cancer can benefit from postoperative chemotherapy. There are several studies evaluating the role of PET scans in monitoring the clinical outcomes of patients with locally advanced pancreatic cancer treated with neoadjuvant treatment[9,33,34]. However, there are very few studies that have evaluated the potential role of preoperative PET scan in predicting the oncologic benefits of postoperative adjuvant chemotherapy in resectable pancreatic cancer. Our results suggest that preoperative PET scans can provide important data for decision for postoperative adjuvant chemotherapy after radical pancreatectomy in resectable pancreatic cancer.

Since the goal of this study was to assess feasibility of qualitatively determined clinical PET type by surgeons, patient population only included those under evaluation for operation. Therefore, the results of current study have limitation in application to unresectable patients. However, according to our clinical experiences of unresectable pancreatic cancer, most cases seem to belong to K-type, suggesting aggressive tumor biology. Further studies based on a larger population including unresectable cases are needed to confirm this observation.

This study is a retrospective study design harboring unavoidable selection bias because not all patients underwent preoperative PET and some patients with neoadjuvant treatment were excluded. In addition, PET parameters, especially SUV_max_, can be influenced by tumor size [35,36]. Therefore, it might be difficult to discriminate between K-type and Non-K-type in small pancreatic cancers, and our data supports this problem. This study showed that pancreatic cancer with disagreement in determination of clinical PET type was significantly smaller than the agreed cases (1.8 ± 0.3 cm vs. 2.5 ± 0.7 cm, *P* = 0.002). When analyzing patients with a radiologic tumor size greater than 2 cm, the average agreement rate increased to 93.3% with a mean pairwise Cohen’s Kappa value of 0.822. Finally, we may not be able to apply clinical PET type to all patients because there are also some clinical conditions that need to be considered, such as impaired renal function[37] and dehydration. ^18^F-FDG is excreted through urine. Therefore, renal function plays an important role in ^18^F-FDG metabolism. In patients with renal impairment, insulin-mediated glucose metabolism is also reduced because of insulin resistance [38]. This may influence FDG uptake in tissues. Accordingly, Torihara et al. [37] have reported that patients with renal dysfunction showed higher physiological FDG uptake in the soft tissue, spleen and blood pool. Despite the general assumption that impaired renal function would influence the distribution and metabolism of ^18^F-FDG, recent report by Akers et al.[8] has shown that impaired renal function does not influence clearance of background activity of ^18^F-FDG PET imaging. Minamimoto et al. [38] have also reported that suspected renal failure will not have a significant influence on assessment of PET imaging. In spite of these findings, FDG uptake in renal calyx is decreased in patients with impaired renal function because of reduced urine activity. Intense FDG uptake might not be seen even in the renal calyx due to the amount of urinary flow at the moment the image was taken. In those cases, the clinical PET type needs to be determined by anecdotal clinician’s memory of the usual intensity of FDG uptake in the renal calyx. Our study results did not include patients with impaired renal function. Limitations exist in determining clinical PET type for patients with abnormal renal functions, however our results have shown that with normal serum Cr levels, mild to moderate decrease in eGFR does not correlate with SUV_max_. Nevertheless, clinical information regarding renal function should be considered when applying clinical PET type.

In conclusion, the current results suggest the clinical feasibility of surgeons’ determined clinical PET type as alternative prognostic marker in resectable pancreatic cancer. True reliability and oncologic significance of clinical PET type need to be reassessed based on a prospective cohort of a large number of patients with resectable pancreatic cancer.

## Supporting information

S1 Data Available data. (XLS)
